# Comparison of the prognosis of symptomatic cerebral infarction and pulmonary embolism in patients with advanced non‐small cell lung cancer

**DOI:** 10.1002/cam4.5647

**Published:** 2023-01-27

**Authors:** Ryota Nakamura, Tadaaki Yamada, Satomi Tanaka, Aosa Sasada, Shinsuke Shiotsu, Nozomi Tani, Takayuki Takeda, Yusuke Chihara, Soichi Hirai, Yoshizumi Takemura, Akihiro Yoshimura, Kenji Morimoto, Masahiro Iwasaku, Shinsaku Tokuda, Young Hak Kim, Koichi Takayama

**Affiliations:** ^1^ Department of Pulmonary Medicine, Graduate School of Medical Science Kyoto Prefectural University of Medicine Kyoto Japan; ^2^ Department of Respiratory Medicine Japanese Red Cross Kyoto Daiichi Hospital Kyoto Japan; ^3^ Department of Pulmonary Medicine Japan Community Health care Organization Kyoto Kuramaguchi Medical Center Kyoto Japan; ^4^ Department of Respiratory Medicine Japanese Red Cross Kyoto Daini Hospital Kyoto Japan; ^5^ Department of Respiratory Medicine Uji‐Tokushukai Medical Center Kyoto Japan; ^6^ Department of Pulmonary Medicine Otsu City Hospital Shiga Japan

**Keywords:** cerebral infarction, ischemic stroke, non‐small cell lung cancer, pulmonary embolism, thromboembolism

## Abstract

**Background:**

Lung cancer patients face a high risk of thromboembolism (TE), which is considered to be a poor prognostic factor. However, the impact of symptomatic cerebral infarction (CI) and pulmonary embolism (PE) on the prognosis of advanced non‐small cell lung cancer (NSCLC) patients is not fully understood.

**Methods:**

We retrospectively identified 46 patients with advanced NSCLC who developed symptomatic CI or PE at five hospitals in Japan between January 2010 and December 2019. Prognosis and biomarker levels after incident CI and PE were investigated.

**Results:**

Of the 46 patients, 36 developed symptomatic CI, and 10 developed symptomatic PE. The median follow‐up duration after incident CI and PE was 18.2 months. Although the proportion of Common Terminology Criteria for Adverse Events grade 4 tended to be higher in patients with PE than in those with CI (30% vs. 11%, *p* = 0.16), the overall survival (OS) after incident TE tended to be worse in patients with CI than in those with PE (median 2.3 months vs. 9.1 months, log‐rank test *p* = 0.17). Multivariate analysis showed that OS after CI was worse in patients with high D‐dimer (DD) levels than in those with low DD levels at the time of incident CI (median 1.3 months vs. 8.3 months, log‐rank *p* < 0.001).

**Conclusions:**

This retrospective study demonstrated that the prognosis of patients tended to be poorer after CI than after PE. The DD levels at the time of incident CI might be a promising predictor of clinical outcomes in advanced NSCLC patients who develop CI.

## Graphical abstract

We retrospectively analyzed the prognosis and predictors of survival in patients with advanced non‐small cell lung cancer who developed symptomatic thromboembolism. In this study, the prognosis of patients with cerebral infarction tended to be poorer than that of patients with pulmonary embolism. In addition, we showed that D‐dimer levels were a promising prognostic predictor in patients who developed cerebral infarction.

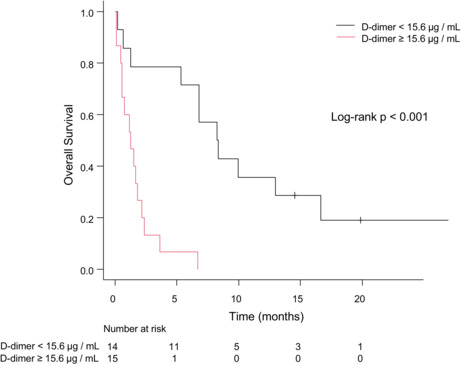

## 1 INTRODUCTION

Lung cancer is the leading cause of cancer‐related deaths worldwide.^1^ Thromboembolism (TE) is the second leading cause of cancer‐related mortality, followed by cancer progression.^2^ It is well known that lung cancer patients are at high risk for TE. Furthermore, TE is considered to be a poor prognostic factor in lung cancer patients. A previous study reported that lung cancer patients with TE had a shorter overall survival (OS) than those without TE.^3^

A retrospective cohort study revealed that lung cancer patients had a 10‐fold increase in venous thromboembolism (VTE) compared to those without cancer; lung cancer patients were at the highest risk of VTE among patients with solid tumors, and VTE was closely related to the incidence of pulmonary embolism (PE).^4^ The incidence of cancer‐associated VTE is increasing due to the prolonged survival of cancer patients, and the increased use of thrombogenic agents such as anti‐angiogenic antibodies.^5^

Cerebral infarction (CI) is a common thromboembolic complication in lung cancer patients.^6^ A retrospective observational study showed that CI occurred in 2.9% of advanced non‐small cell lung cancer (NSCLC) patients, and advanced NSCLC patients who developed CI had a median survival time of only 36 days.^7^

The prognosis of advanced NSCLC patients with TE is generally poor, and it is critical to develop predictive biomarkers for survival outcomes. However, few studies have focused on advanced NSCLC patients with TE, especially those with CI.

Therefore, this study aimed to determine the prognosis and identify potential predictors of survival in advanced NSCLC patients who developed symptomatic CI and PE in a real‐world setting.

## 2 METHODS

### 2.1 Patients

In this study, we retrospectively enrolled patients with advanced NSCLC who developed Common Terminology Criteria for Adverse Events (CTCAE) grade 2 (mild to moderate neurologic deficit; limiting instrumental Activities of Daily Living [ADL]) and more severe CI or CTCAE grade 3 (urgent medical intervention indicated) and more severe PE at five hospitals in Japan between January 2010 and December 2019 by reviewing medical records. We gathered the following data: sex, age at incident CI and PE, smoking history, history of stroke, history of antithrombotic drugs usage, body mass index at incident CI and PE, laboratory results at incident CI and PE, Eastern Cooperative Oncology Group Performance Status (ECOG‐PS) before incident CI and PE, TNM stage based on classification system version 8, presence of brain metastasis, liver metastasis, pleural effusion, and double cancer; histological subtype, presence of epidermal growth factor receptor (EGFR) mutation; programmed death‐ligand 1 expression level in tumors, radiation history, treatment line, treatment for cancer; TE type, severity classified using the CTCAE version 5.0, treatment for CI and PE; and OS after incident CI and PE. The Ethics Committees of the Kyoto Prefectural University of Medicine (approval no.: ERB‐C‐1733‐4), and each participating hospital approved the study protocol.

### 2.2 Statistical analysis

Data were collected retrospectively in June 2020. The median follow‐up duration after incident CI and PE was 18.2 months. During the follow‐up period, we identified 38 deaths out of 46 patients. The Mann–Whitney U test was used to compare the means of the continuous variables. The Fisher's exact test was used to compare the proportions of categorical variables. The Kaplan–Meier method was used to calculate survival curves, and the log‐rank test was used to compare the differences. When comparing OS after incident CI and PE in patients with CI and PE, we performed propensity‐score matching using the following variables: sex, age, ECOG‐PS, and presence of treatment for cancer. Nearest neighbor matching was performed at a ratio of 1:1. Caliper was set at 0.2. Hazard ratios and 95% confidence intervals were calculated using Cox proportional hazards models. Patients who were alive were censored on the date of the last follow‐up. The cutoff values of platelet count, lactate dehydrogenase, albumin, C‐reactive protein, and D‐dimer (DD) levels were set at the respective median value. Because the cutoff value of DD levels as a prognostic factor remained unclear, we performed receiver operating characteristic (ROC) curve analysis to identify the optimal cutoff value for DD. In the ROC analysis, the sum of specificity and sensitivity for 30‐days mortality was maximized at 16.1 μg/mL. Therefore, we considered that it is suitable for the median value of 15.6 μg/mL as a reasonable cutoff value for DD (Figure S1). Patients whose date of death was missing were excluded from survival analysis. Patients with missing laboratory data were excluded from analysis. EZR statistical software version 1.42 was used for statistical analysis.^8^ Statistical significance was set at *p* < 0.05. All statistical tests were two‐tailed.

## 3 RESULTS

### 3.1 Patient characteristics and laboratory findings

We identified 46 patients with advanced NSCLC who developed symptomatic CI or PE. Of these, 36 and 10 patients developed CI and PE, respectively.

The characteristics of the patients classified according to the TE type are shown in Table 1. Patients with CI and PE had a similar median age of 71 years with ranges, 58–86 years, and 49–77 years, respectively. A total of 23 (63.4%) patients with CI and seven (70%) with PE were being treated with anticancer drugs at incident CI and PE. The body mass index at incident CI and PE was significantly higher in patients with CI than in those with PE (*p* = 0.033). The proportion of patients with pleural effusion at incident CI and PE was higher in the PE group than in the CI group (60% vs. 16.7%, *p* = 0.0061).

**TABLE 1:** Patient characteristics.

Characteristics	Total (%)	PE (%)	CI (%)	*p*‐value
Number	46	10	36	
Age				
Median (range)	71 (49–86)	71 (49–77)	71 (58–86)	0.44
Sex				
Male	30 (65.2)	8 (80.0)	22 (61.1)	0.46
Female	16 (34.8)	2 (20.0)	14 (38.9)	
ECOG‐performance status				
0	11 (23.9)	1 (10.0)	10 (27.8)	0.11^a^
1	17 (37.0)	7 (70.0)	10 (27.8)	
≥2	17 (37.0)	2 (20.0)	15 (41.6)	
Missing data	1 (2.2)	0 (0.0)	1 (2.8)	
Smoking status				
Current/Former	30 (65.2)	5 (50.0)	25 (69.4)	0.28
Never	16 (34.8)	5 (50.0)	11 (30.6)	
Body mass index				
Median (range)	20.3 (11.9–29.0)	19.9 (27.8–14.8)	22.2 (11.9–29.0)	0.033
History of brain stroke				
Yes	11 (23.9)	1 (10.0)	10 (27.8)	0.41
No	35 (76.1)	9 (90.0)	26 (72.2)	
Histology				
Adenocarcinoma	40 (87.0)	10 (100)	30 (83.3)	1.0^b^
Squamous cell carcinoma	3 (6.5)	0 (0.0)	3 (8.3)	
Other	3 (6.5)	0 (0.0)	3 (8.3)	
Oncogenic driver				
EGFR mutation positive	17 (37.0)	6 (60.0)	11 (30.6)	0.27^c^
EGFR wild type	28 (60.9)	4 (40.0)	24 (66.7)	
Not investigated	1 (2.2)	0 (0.0)	1 (2.8)	
PD‐L1 TPS				
≥50%	8 (17.4)	2 (20.0)	6 (16.7)	1.0^d^
1–49%	5 (10.9)	2 (20.0)	3 (8.3)	
<1%	4 (8.7)	1 (10.0)	3 (8.3)	
Not investigated	29 (63.0)	5 (50.0)	24 (66.6)	
Stage				
IIIB/IV	41 (89.1)	8 (80.0)	33 (91.7)	0.30
Recurrent	5 (10.9)	2 (20.0)	3 (8.3)	
Brain metastasis				
Yes	35 (76.1)	9 (90.0)	26 (72.2)	0.41
No	11 (23.9)	1 (10.0)	10 (27.8)	
Liver metastasis				
Yes	8 (17.4)	0 (0.0)	8 (22.2)	0.17
No	38 (82.6)	10 (100)	28 (77.8)	
Pleural effusion				
Yes	12 (26.1)	6 (60.0)	6 (16.7)	0.0061
No	34 (73.9)	4 (40.0)	30 (83.3)	
Treatment for cancer				
No treatment	16 (34.8)	3 (30.0)	13 (36.1)	1.0^e^
Before starting treatment	10 (21.7)	2 (20.0)	8 (22.2)	
BSC	6 (13.0)	1 (10.0)	5 (13.9)	
With treatment	30 (65.2)	7 (70.0)	23 (63.9)	
EGFR‐TKI	9 (19.6)	3 (30.0)	6 (16.7)	
ICI	1 (2.2)	0 (0.0)	1 (2.8)	
Cytotoxic drug	15 (32.6)	3 (30.0)	12 (33.3)	
Cytotoxic drug + Bevacizumab	4 (8.7)	0 (0.0)	4 (11.1)	
Cytotoxic drug + Bevacizumab + ICI	1 (2.2)	1 (10.0)	0 (0.0)	
Treatment line				
1st	14 (30.4)	4 (40.0)	10 (27.8)	
2nd	5 (10.9)	1 (10.0)	4 (11.1)	
3rd	5 (10.9)	1 (10.0)	4 (11.1)	
4th–	6 (13.0)	1 (10.0)	5 (13.9)	
Radiation history				
Yes	10 (21.7)	2 (20.0)	8 (22.2)	1.0
No	36 (78.2)	8 (80.0)	28 (77.8)	
Double cancer				
Positive	1 (2.2)	1 (10.0)	0 (0.0)	0.080
Negative	45 (97.8)	9 (90.0)	36 (100)	

Abbreviations: BSC, best supportive care; CI, cerebral infarction; ECOG, eastern cooperative oncology group; EGFR, epidermal growth factor receptor; ICI, immune checkpoint inhibitor; PD‐L1 TPS, programmed death ligand 1 tumor proportion score; PE, pulmonary embolism; TKI, tyrosine kinase inhibitor.

^a^ ECOG‐performance status 0/1 versus 2/3.

^b^ Adenocarcinoma versus all others.

^c^ EGFR mutation positive versus all others.

^d^ PD‐L1 TPS ≥ 50% versus all others.

^e^ With treatment versus without treatment.

The laboratory data classified by TE type are presented in Table 2. The platelet count was significantly higher in patients with PE than in those with CI (*p* = 0.030). The median DD level was high in patients with both CI and PE (CI and PE, 15.6 and 29.3 μg/mL, respectively).

**TABLE 2:** Laboratory data.

Median (interquartile range)	PE	CI	*p*‐value
Platelet (×10^4^/μL)	24.0 (18.6–29.7)	16.4 (10.0–25.2)	0.030
CRP (mg/dL)	2.35 (0.49–5.04)	5.01 (1.38–8.00)	0.29
LDH (IU/L)	335 (252–394)	321 (258–459)	0.95
Albumin (mg/dL)	3.40 (2.83–3.71)	3.05 (2.60–3.70)	0.37
D‐dimer (μg/mL)	29.3 (9.5–38.3)	15.6 (3.94–36.61)	0.33

Abbreviations: CI, cerebral infarction; CRP, C‐reactive protein; LDH, lactate dehydrogenase; PE, pulmonary embolism.

### 3.2 Clinical characteristics of CI and PE

**FIGURE 1:**
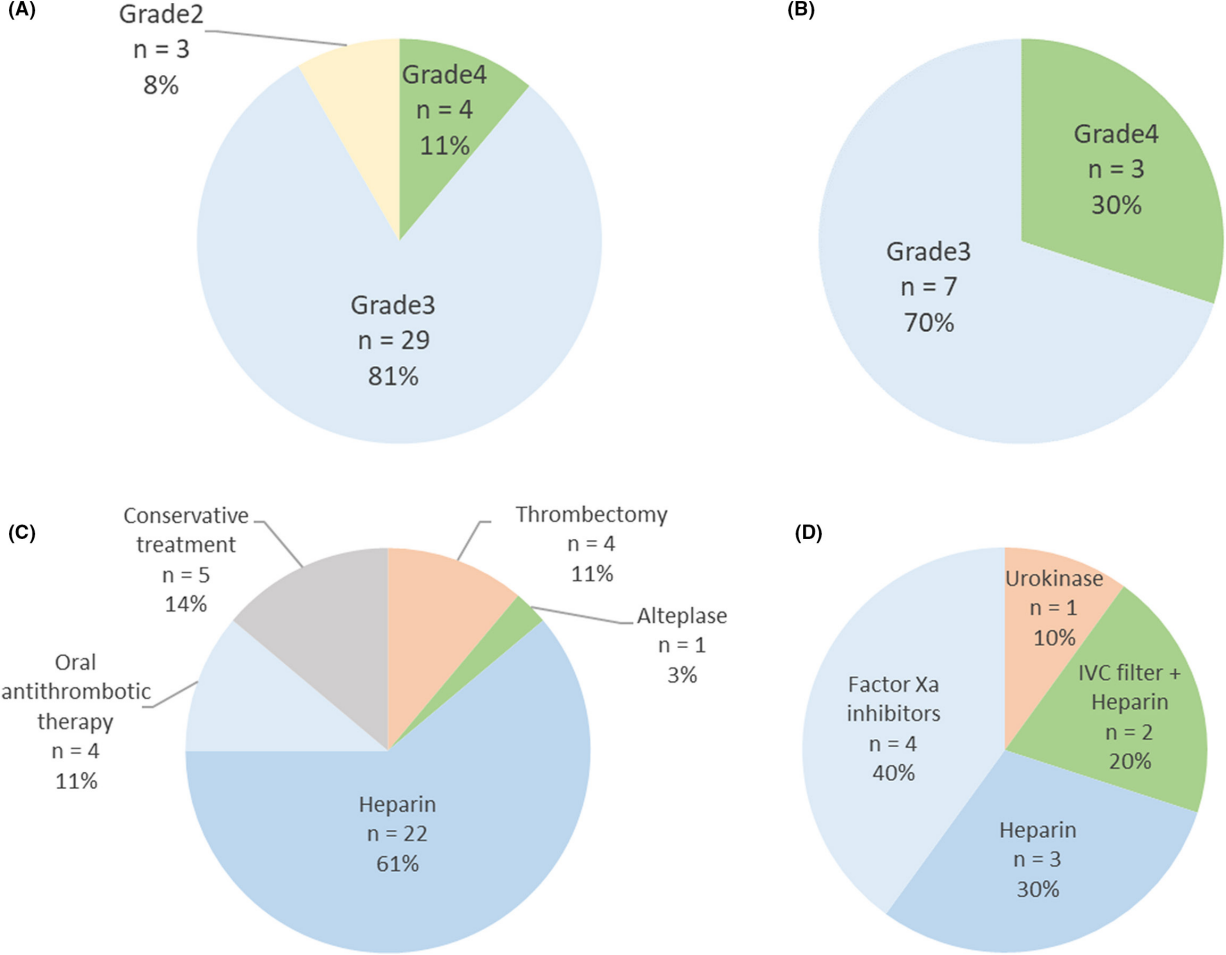
CTCAE grades for CI (A) and PE (B). Acute management for CI (C) and PE (D). CI, cerebral infarction; CTCAE, common terminology criteria for adverse events; IVC, inferior vena cava; PE, pulmonary embolism.

The CTCAE grades in patients with CI and PE are shown in Figure 1A,B, respectively. Among 36 patients with CI, four (11%) had grade 4 CI, 29 (81%) had grade 3 CI, and three (8%) had grade 2 CI. Among 10 patients with PE, three (30%) had grade 4 PE, whereas seven (70%) had grade 3 PE. Acute management of CI and PE are shown in Figure 1C,D, respectively. Among 36 patients with CI, four (11%), one (3%), 26 (72%), and five (14%) received mechanical thrombectomy, thrombolysis therapy with alteplase, anticoagulant therapy with heparin or oral anticoagulants, and conservative treatment, respectively. Among four patients receiving oral anticoagulants, two received antiplatelet agents, and one each received factor Xa inhibitors and warfarin. Among 10 patients with PE, one (10%), two (20%), and seven (70%) received thrombolysis therapy with urokinase, inferior vena cava filter placement, and anticoagulant therapy with heparin or factor Xa inhibitors, respectively.

**FIGURE 2:**
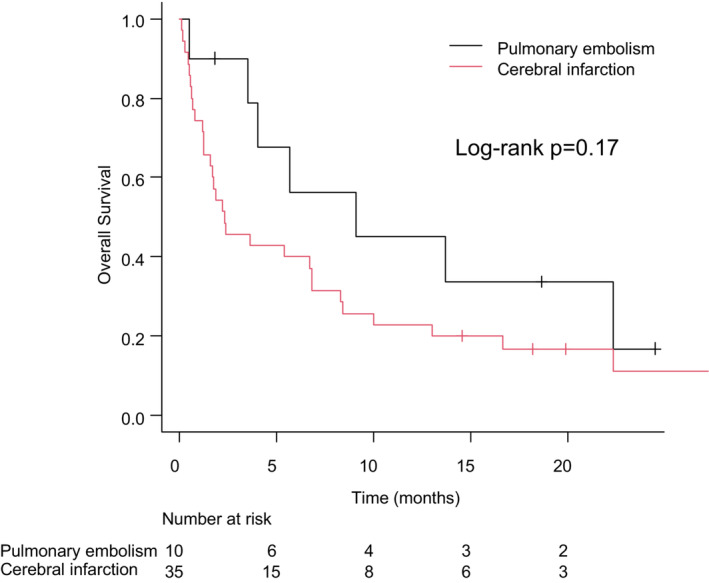
Kaplan–Meier curves for OS after incident CI and PE according to the types of TE. CI, cerebral infarction; OS, overall survival; PE, pulmonary embolism; TE, thromboembolism.

The Kaplan–Meier curves for OS after incident CI and PE are shown in Figure 2. OS after incident CI and PE tended to be worse in patients with CI than in those with PE (median 2.3 months vs. 9.1 months, log‐rank test *p* = 0.17). To control for confounding variables, we performed the propensity‐score analysis, which showed no significant difference in OS after incident CI and PE between patients with CI and those with PE (median 8.4 months vs. 11.4 months, log‐rank test *p* = 0.90) (Figure S2).

### 3.3 Prognostic factor for CI and PE events

The results of univariate analyses for OS after incident CI and PE are shown in Table 3 and Table S1, respectively. In patients with CI, univariate analysis demonstrated that OS was significantly associated with antithrombotic drug use, platelet count, C‐reactive protein, lactate dehydrogenase, and DD levels. In patients with PE, univariate analysis showed that OS was significantly associated with serum albumin level ≥3.05 mg/dL. In patients with CI, multivariate analysis for OS showed that DD level ≥ 15.6 μg/mL was significantly associated with poor prognosis (hazard ratio, 8.33; 95% confidence interval, 2.57–27.1; *p* < 0.001).

**TABLE 3:** Cox proportional hazards models for OS in patients with CI. (A) Univariate analysis and (B) multivariate analysis. (*n* = 35).

(A)		
Items	OS (Univariate analysis)	
	HR (95% confidence interval)	*p*‐value
Age		
≥75	0.87 (0.40–1.91)	0.73
Sex		
Female	0.62 (0.29–1.33)	0.22
ECOG‐PS		
≥2	1.24 (0.60–2.55)	0.56
CTCAE		
≥Grade 4	0.92 (0.32–2.66)	0.88
Smoking status		
Current/Former	1.19 (0.54–2.60)	0.67
BMI		
≥25 kg/m^2^	1.88 (0.44–8.11)	0.40
History of brain stroke		
Yes	1.21 (0.55–2.64)	0.64
Antithrombotic drug		
Yes	2.40 (1.03–5.56)	0.042
Histology		
Adenocarcinoma	0.82 (0.28–2.36)	0.71
EGFR mutation		
Positive	0.64 (0.29–1.42)	0.27
Postoperative recurrence		
Yes	1.84 (0.54–6.24)	0.33
Brain metastasis		
Yes	0.39 (0.15–1.03)	0.057
Liver metastasis		
Yes	1.35 (0.57–3.18)	0.49
Pleural effusion		
Yes	1.08 (0.44–2.65)	0.87
Platelet		
≥16.4 × 10^4^/μL	0.08 (0.03–0.26)	< 0.001
CRP		
≥5.01 mg/dL	6.46 (2.51–16.7)	< 0.001
LDH		
≥321 IU/L	3.58 (1.62–7.95)	0.002
Albumin		
≥3.05 mg/dL	0.47 (0.20–1.10)	0.081
D‐dimer		
≥15.6 μg/mL	8.29 (2.56–26.9)	< 0.001

(B)		
Items	OS (Multivariate analysis)	
	HR (95% confidence interval)	*p*‐value
ECOG PS ≥ 2	1.05 (0.48–2.32)	0.90
CTCAE grade ≥ 4	0.94 (0.27–3.31)	0.93
D‐dimer ≥ 15.6 μg/mL	8.33 (2.57–27.1)	<0.001

Abbreviations: BMI, body mass index; CRP, C‐reactive protein; CTCAE, common terminology criteria for adverse events; ECOG, eastern cooperative oncology group; EGFR, epidermal growth factor receptor; HR, hazard ratio; LDH, lactate dehydrogenase; OS, Overall survival; PS, performance status.

**FIGURE 3:**
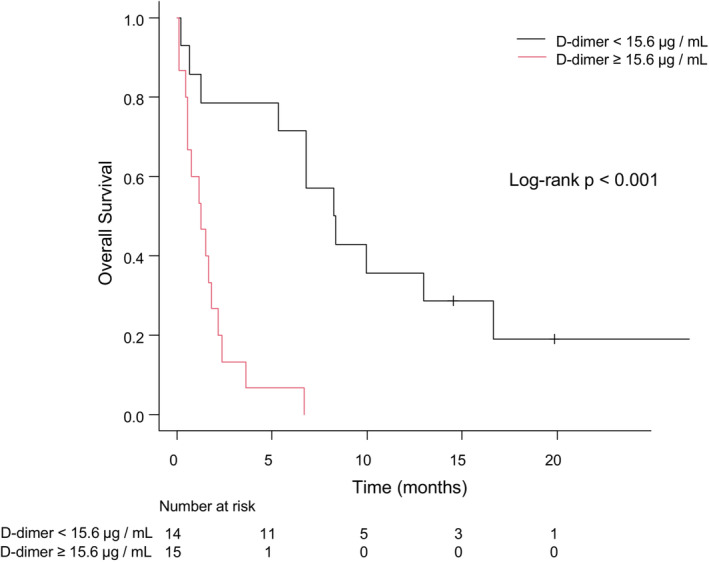
Kaplan–Meier curves for OS after incident CI according to the D‐dimer level. CI, cerebral infarction; OS, overall survival.

The Kaplan–Meier curves illustrated that patients with high DD levels had significantly shorter OS after incident CI than those with low DD levels (median 1.3 vs. 8.3 months, log‐rank *p* < 0.001) (Figure 3).

## 4 DISCUSSION

Accumulating evidence regarding complications indicates that lung cancer patients are at high risk for TE, which is related to poor prognosis.^3^, ^4^, ^6^, ^7^ However, the impact of symptomatic TE on the prognosis of advanced NSCLC patients remains unclear. In this study, we identified high serum DD levels at the time of onset as a poor prognostic factor in advanced NSCLC patients who developed symptomatic CI. A previous meta‐analysis demonstrated that DD levels predicted in‐hospital mortality in patients with stroke.^9^ Moreover, DD level was reported to predict 30‐day mortality in cancer patients who developed CI.^10^, ^11^ In this study, NSCLC patients with high DD levels had a significantly shorter survival period after incident CI than those with low DD levels. This is the first study to demonstrate the significance of the DD level as a potential prognostic predictor in advanced NSCLC patients who develop symptomatic CI. Interestingly, in a previous study, high DD levels were associated with embolic signals assessed using transcranial Doppler in cancer patients who developed CI, suggesting that the DD level might be a surrogate marker of hypercoagulability in cancer patients.^12^

In this study, the proportion of patients with severe symptoms with CTCAE grade 4 tended to be higher in patients with PE than those with CI, whereas those with CI tended to have a poorer prognosis than those with PE. Disseminated intravascular coagulation predicts an extremely poor prognosis for lung cancer patients with TE.^3^ In our study, patients with CI had a significantly lower platelet count than those with PE, whereas DD levels were increased in both groups. Based on these results, patients with CI may be more likely to develop disseminated intravascular coagulation than those with PE. Furthermore, patients with lung cancer‐associated CI have more severe neurological defects than those with non‐cancer‐associated CI, suggesting that it might worsen the performance status and lead to discontinuation of systemic chemotherapy.^13^ However, the propensity score analysis revealed that there was no significant difference in OS after incident TE between patients with CI and those with PE. Regarding patient characteristics, the proportion of patients with poor performance status tended to be higher in patients with CI. These observations suggested that patients with CI might have a poorer prognosis due to their poorer general condition compared to those with PE.

Although most patients who develop symptomatic CI receive anticoagulant therapy, the prognosis is poor, especially in patients with high DD levels. Therefore, it is necessary to develop a novel strategy to manage symptomatic CI. In recent years, mechanical thrombectomy has been established as the standard treatment for patients with large‐vessel occlusion‐related CI.^14^ In this study, among four patients who underwent mechanical thrombectomy, one patient survived for 22.6 months after incident CI, indicating that mechanical thrombectomy might be a promising therapy for large‐vessel occlusion‐related CI in cancer patients. Therefore, further investigations are needed to validate the efficacy and safety of mechanical thrombectomy in advanced NSCLC patients who develop large‐vessel occlusion‐related CI.

Our study had some limitations. First, the number of patients with symptomatic CI and PE was small, suggesting that there might be under‐estimation in observation. In this study, the number of patients with symptomatic PE was smaller than that of symptomatic CI. A previous prospective study of patients with solid tumors had reported a small proportion of PE among VTE, and a low frequency of symptomatic VTE, suggesting that it might be the reason for the low number of patients with symptomatic PE in our study.^15^ Second, there may have been biases, such as selection and information, due to the retrospective nature of the study, although we conducted a multicenter study. Third, we excluded patients with asymptomatic CI and PE; therefore, it was unknown whether the findings in this study could be applied to patients with asymptomatic CI and PE. Fourth, we could not examine the clinical course of advanced NSCLC patients without CI and PE.

## 5 CONCLUSIONS

We retrospectively analyzed the prognosis and potential predictors of survival in advanced NSCLC patients who developed symptomatic CI and PE in a real‐world setting. In this study, the prognosis of NSCLC patients with symptomatic CI tended to be poorer than that of patients with symptomatic PE. Furthermore, we demonstrated that DD levels were a promising prognostic predictor in advanced NSCLC patients who developed symptomatic CI. Therefore, it is necessary to develop better management strategies for advanced NSCLC patients who develop symptomatic CI, especially for those with high DD levels at the time of onset. Because the findings of this study were obtained from a limited number of cases with CI and PE, it is warranted to validate them using larger prospective cohorts.

## AUTHOR CONTRIBUTIONS

**Ryota Nakamura:** Data curation (lead); formal analysis (lead); methodology (equal); writing – original draft (equal). **Tadaaki Yamada:** Conceptualization (lead); methodology (equal); project administration (lead); writing – original draft (equal). **Satomi Tanaka:** Conceptualization (supporting); resources (equal). **Aosa Sasada:** Resources (equal). **Shinsuke Shiotsu:** Resources (equal). **Nozomi Tani:** Resources (equal). **Takayuki Takeda:** Resources (equal). **Yusuke Chihara:** Resources (equal). **Soichi Hirai:** Resources (equal). **Yoshizumi Takemura:** Resources (equal). **Akihiro Yoshimura:** Writing – review and editing (equal). **Kenji Morimoto:** Writing – review and editing (equal). **Masahiro Iwasaku:** Writing – review and editing (equal). **Shinsaku Tokuda:** Writing – review and editing (equal). **Young Hak Kim:** Writing – review and editing (equal). **Koichi Takayama:** Supervision (lead).

## CONFLICT OF INTEREST

TY received commercial research grants from Pfizer, Ono Pharmaceutical, Janssen Pharmaceutical K.K., AstraZeneca, and Takeda Pharmaceutical Company Limited and personal fees from Eli Lilly. KT received research grants from Chugai‐Roche and Ono Pharmaceutical and personal fees from AstraZeneca, Chugai‐Roche, MSD‐Merck, Eli Lilly, Boehringer‐Ingelheim, and Daiichi‐Sankyo. The other authors have no conflicts of interest to declare.

## ETHICAL APPROVAL STATEMENT

The Ethics Committee of the Kyoto Prefectural University of Medicine authorized the study protocol (ERB‐C‐1733‐4), and the study was carried out in compliance with the Declaration of Helsinki on the handling of patients' personal data.

## PATIENT CONSENT STATEMENT

Owing to the retrospective nature of the study, the requirement for informed consent was waived, and the Ethics Committee of each individual hospital accepted the use of the official website to offer an opt‐out option.

## CLINICAL TRIAL REGISTRATION

Not applicable.

## Supporting information


Figure S1.

Figure S2.

Table S1.

## Data Availability

The datasets are available from the corresponding author, TY, upon justifiable request.
