# Supplementary figures and images for: Comparison of the prognosis of symptomatic cerebral infarction and pulmonary embolism in patients with advanced non‐small cell lung cancer
Source: Cancer Med. 2023 Jan 27;12(8):9097–105. doi: 10.1002/cam4.5647 (PMC10166976; doi:10.1002/cam4.5647)

## Slide 1
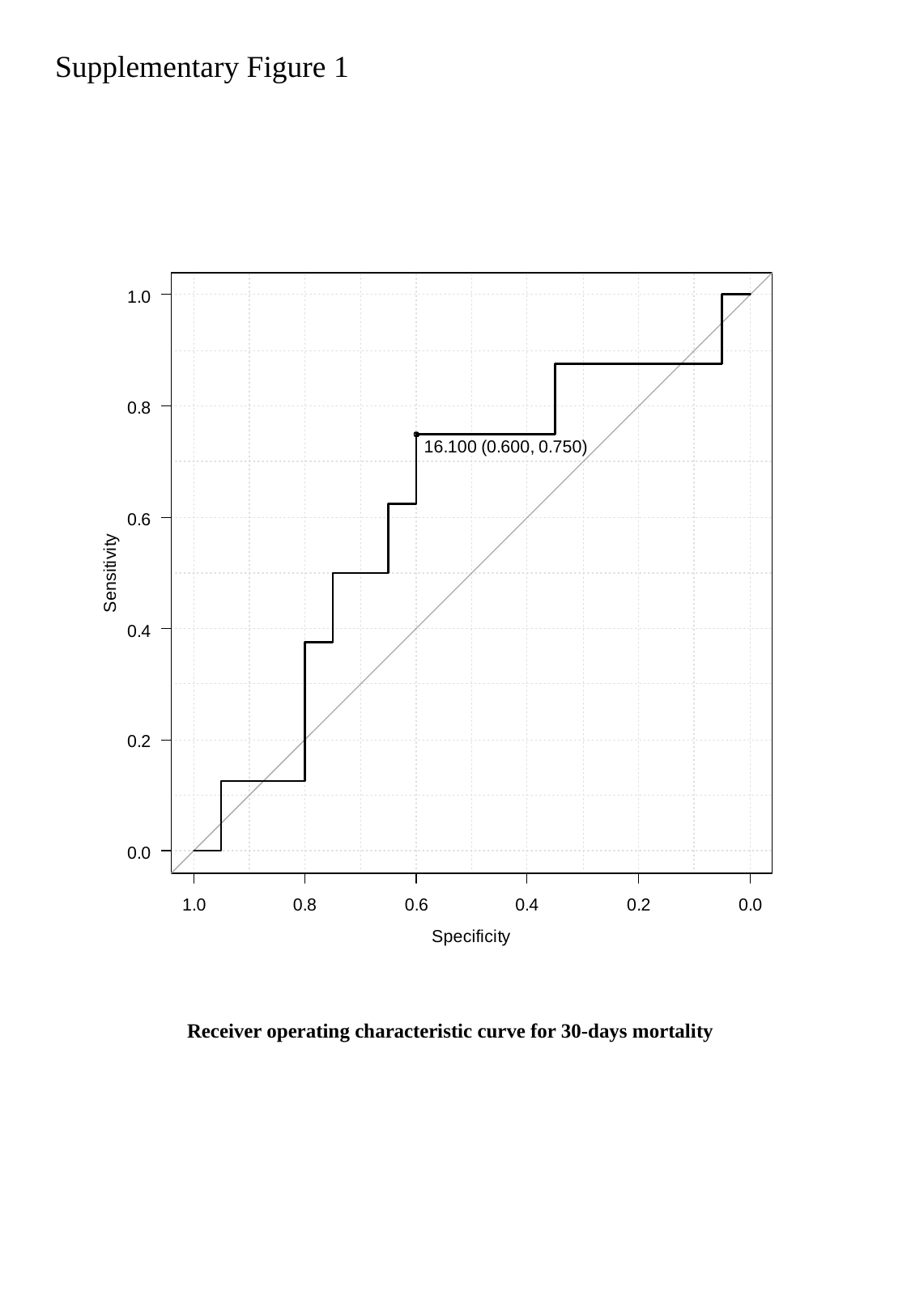

Supplementary Figure 1
Receiver operating characteristic curve for 30-days mortality

Supplement: Supplementary file 1 — Figure S1. [file CAM4-12-9097-s003.pptx]
