# Supplementary material for: Comparison of the prognosis of symptomatic cerebral infarction and pulmonary embolism in patients with advanced non‐small cell lung cancer
Source: Cancer Med. 2023 Jan 27;12(8):9097–105. doi: 10.1002/cam4.5647 (PMC10166976; doi:10.1002/cam4.5647)
Supplement: Supplementary file 2 — Figure S2. [file CAM4-12-9097-s002.pptx]

## Slide 1
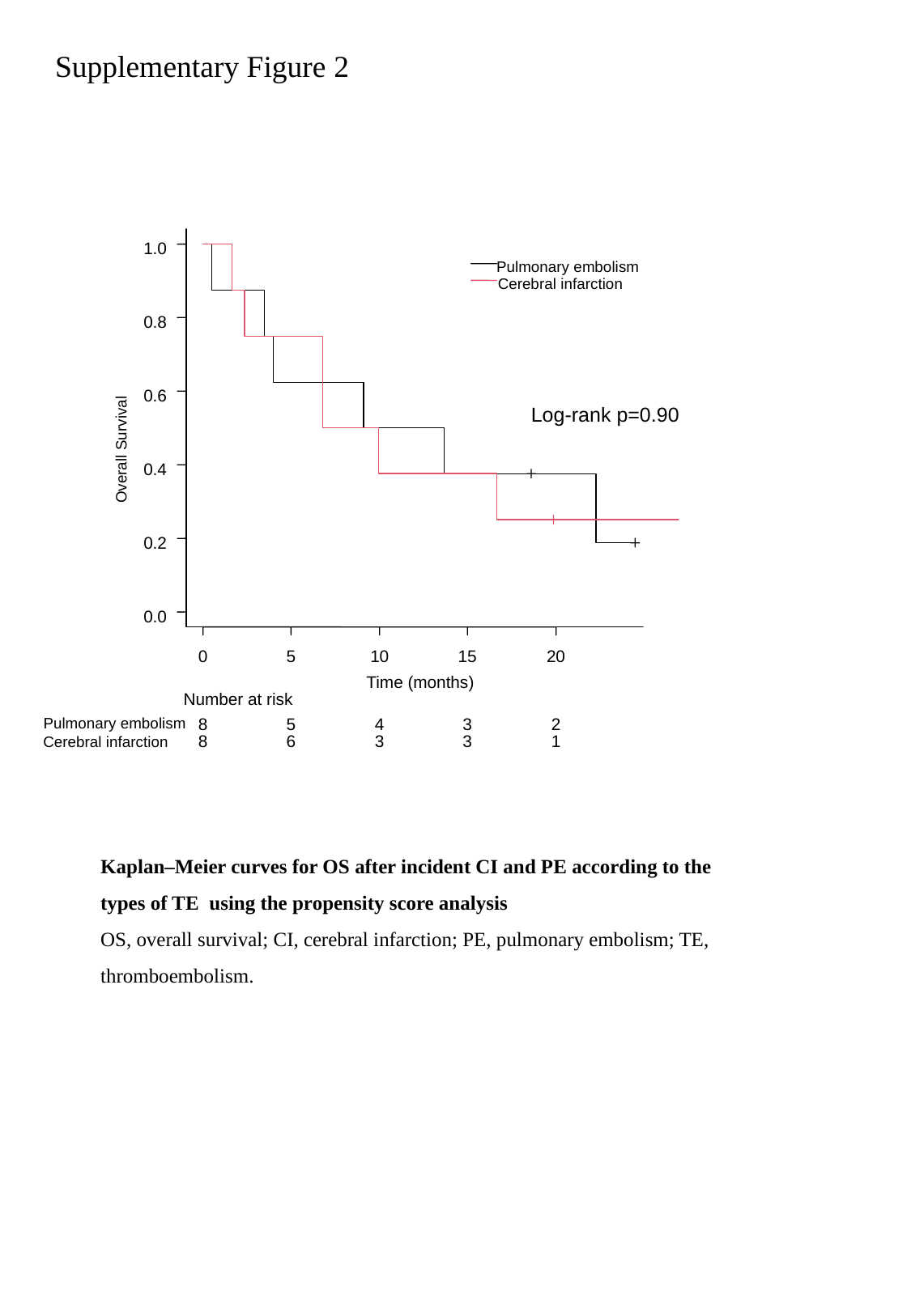

Supplementary Figure 2
1.0
Pulmonary embolism
Cerebral infarction
0.8
0.6
Overall Survival
0.4
0.2
0.0
0
5
10
15
20
Time (months)
Number at risk
Pulmonary embolism
8
5
4
3
2
8
6
3
3
1
Cerebral infarction
Log-rank p=0.90
Kaplan–Meier curves for OS after incident CI and PE according to the types of TE using the propensity score analysis
OS, overall survival; CI, cerebral infarction; PE, pulmonary embolism; TE, thromboembolism.
